# 443. Do people living with HIV lose weight on GLP-1 agonist therapy?

**DOI:** 10.1093/ofid/ofac492.518

**Published:** 2022-12-15

**Authors:** Lamiya Tauhid, Mima Fondong, Aish Lovett, Patricia J Kissinger, Yussef Bennani, Meredith E Clement

**Affiliations:** Louisiana State University Health Sciences Center New Orleans, New Orleans, Louisiana; Louisiana State University Health Science Center New Orleans, New Orleans, Louisiana; Louisiana State University Health Science Center–New Orleans, New Orleans, Louisiana; Tulane University School of Public Health & Tropical Medicine, New Orleans, Louisiana; LSU Health Sciences Center New Orleans, New Orleans, Louisiana; Louisiana State University Health Science Center–New Orleans, New Orleans, Louisiana

## Abstract

**Background:**

Anti-retroviral therapy (ART) has been associated with significant weight gain and metabolic derangements in persons with HIV (PWH), and many PWH on ART experience comorbid obesity. GLP-1 receptor agonists (GLP-1RA) are used to treat type-2 diabetes and obesity in people without HIV infection, but data on the use of these agents in PWH on ART are limited.

**Methods:**

We extracted data from electronic medical records of PWH on ART receiving care at a clinic in New Orleans, LA who had been started on GLP-1RA therapy. We tracked weight (change in body weight, body mass index [BMI]) and changes in hemoglobin A1c (Hba1c) over time from initiation of GLP-1RA to April 2022. A control group of PWH on metformin only will be compared to those on GLP-1RA at a later stage of analysis.

**Results:**

Of 35 PWH on GLP-1RA, the mean age was 55.2 (standard deviation [SD] 10.0); 29 (83%) were Black/African American, 14 (40%) were assigned female at birth, 21 (60%) were assigned male at birth, and 2 were non-binary. Average BMI was 35.7 (SD 9.8) and average HbA1c was 9.5 (SD 2.6) at baseline. Integrase inhibitors were prescribed for 31 (89%) and metformin was prescribed for 20 (57%). Other baseline characteristics are shown in Figure 1. Mean duration of GLP-1RA therapy was 20.6 months (SD 14.0), and 20 (57%) had greater than 12 months of follow-up. Most (23, 66%) on GLP-1RA lost weight, 3 (9%) had a stable weight, and 9 (26%) gained weight. Five percent or more of body weight was lost by 11 (31%) of the total cohort (those with any amount of time on GLP-1RA) and by 9/20 (45%) of those on GLP-1RA for more than 12 months.

**Figure:**
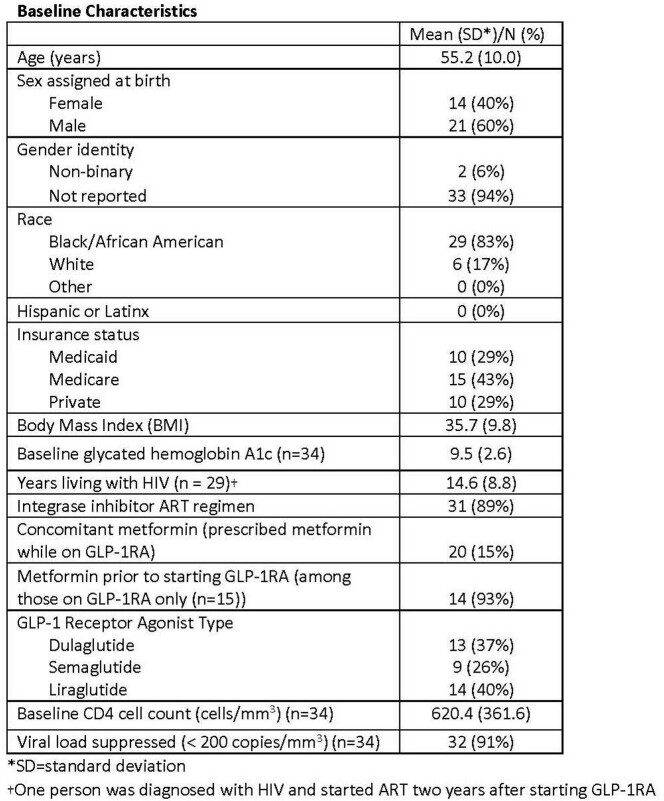

**Conclusion:**

Our data show moderate weight loss among PWH on GLP-1RA. This study is limited by small sample size and limited follow-up time. Further research is needed to determine whether GLP-1RAs are an effective treatment option for obesity in PWH.

**Disclosures:**

**Meredith E. Clement, MD**, Gilead Sciences: Grant/Research Support|Roche: Advisor/Consultant|Viiv Healthcare: Advisor/Consultant|Viiv Healthcare: Grant/Research Support.

